# FUNCTIONAL HEARING AND VISION IMPAIRMENT AND SOCIAL ISOLATION OVER 8 YEARS IN COMMUNITY-DWELLING OLDER ADULTS

**DOI:** 10.1093/geroni/igac059.538

**Published:** 2022-12-20

**Authors:** Alison Huang, George Rebok, Nicholas Reed, Jonathan Suen, Bonnielin Swenor, Thomas Cudjoe, Jennifer Deal

**Affiliations:** Johns Hopkins Bloomberg School of Public Health, Baltimore, Maryland, United States; Johns Hopkins Bloomberg School of Public Health, Baltimore, Maryland, United States; Johns Hopkins Bloomberg School of Public Health, Baltimore, Maryland, United States; Johns Hopkins University, Baltimore, Maryland, United States; Johns Hopkins University, Baltimore, Maryland, United States; Johns Hopkins School of Medicine, Baltimore, Maryland, United States; Johns Hopkins University, Baltimore, Maryland, United States

## Abstract

One in four older adults experience social isolation. Sensory impairment is a potentially modifiable risk factor for social isolation. Little is known about the long-term impact of sensory impairment on social well-being. This study quantifies the longitudinal associations between hearing, vision, and concurrent hearing and vision impairment (dual sensory impairment) and social isolation over 8 years among older adults. Data were from the National Health and Aging Trends Study (NHATS), a nationally representative longitudinal study (2011- 2019) of U.S. Medicare beneficiaries. Social isolation was measured by a composite that incorporated four domains: living arrangement, core discussion network size, religious services attendance, and social participation. Baseline hearing and vision were measured by self-report. Associations between sensory impairments and odds of overall social isolation over 8 years were assessed using multivariate generalized logistic mixed models adjusted for demographic and health characteristics. Among 5,552 participants, 18.9% reported hearing impairment, 4.8% reported vision impairment, and 2.3% reported dual sensory impairment. Over 8 years, hearing impairment was associated with 28% greater odds of social isolation. Specifically, participants with hearing impairment were more likely to live alone and limit engagement in social activities. A similar pattern of association was observed for dual sensory impairment; however, estimates did not reach statistical significance. No association was observed between vision impairment and social isolation. Older adults with hearing impairment may be a clinically important subgroup to monitor for social isolation. Interventions for increasing social support and social participation may be especially valuable for older adults with hearing impairment.

